# The spatiotemporal dynamics of semantic integration in the human brain

**DOI:** 10.1038/s41467-023-42087-8

**Published:** 2023-10-24

**Authors:** Elliot Murphy, Kiefer J. Forseth, Cristian Donos, Kathryn M. Snyder, Patrick S. Rollo, Nitin Tandon

**Affiliations:** 1 Vivian L. Smith Department of Neurosurgery, McGovern Medical School, University of Texas Health Science Center at Houston, Houston, TX 77030 USA; 2 Texas Institute for Restorative Neurotechnologies, University of Texas Health Science Center at Houston, Houston, TX 77030 USA; 3 Faculty of Physics, University of Bucharest, Măgurele 077125 Bucharest, Romania; 4 Memorial Hermann Hospital, Texas Medical Center, Houston, TX 77030 USA

**Keywords:** Language, Reading

## Abstract

Language depends critically on the integration of lexical information across multiple words to derive semantic concepts. Limitations of spatiotemporal resolution have previously rendered it difficult to isolate processes involved in semantic integration. We utilized intracranial recordings in epilepsy patients (n = 58) who read written word definitions. Descriptions were either referential or non-referential to a common object. Semantically referential sentences enabled high frequency broadband gamma activation (70–150 Hz) of the inferior frontal sulcus (IFS), medial parietal cortex, orbitofrontal cortex (OFC) and medial temporal lobe in the left, language-dominant hemisphere. IFS, OFC and posterior middle temporal gyrus activity was modulated by the semantic coherence of non-referential sentences, exposing semantic effects that were independent of task-based referential status. Components of this network, alongside posterior superior temporal sulcus, were engaged for referential sentences that did not clearly reduce the lexical search space by the final word. These results indicate the existence of complementary cortical mosaics for semantic integration in posterior temporal and inferior frontal cortex.

## Abstract (web summary)

Limitations of spatiotemporal resolution have rendered it difficult to isolate language. Here, intracranial recordings were used to map semantic processes pertaining to sentence integration, unveiling complementary roles for frontotemporal brain regions.

## Introduction

The neurobiology of human sentence processing brings with it major implications for our understanding of the organization and timing of cortical computation. It still remains unclear whether multiple cortical regions are needed to evaluate sentence meaning^1,2^, or if highly overlapping heterogeneous constituents within a single region can encode distinct aspects of meaning^3^. Retrieving specific words from memory to refer to objects in the world is an essential contribution made by language to our cognitive abilities^4–12^. The fundamentally generative nature of language enables us to derive concepts using cues we may never have encountered, such as when the phrase “an elongated yellow fruit” is immediately recognized as referring to a banana. Such derivations depend upon the rapid integration of multiple lexical objects into a larger structure, but this crucially occurs alongside other semantic processes^13,14^. We know broadly that language-related semantic processes engage the posterior temporal lobe^15–17^ and prefrontal and parietal cortices^18–20^, but there remains no general consensus in the literature. Much work has focused on picture naming (overt reference) rather than naming to a definition (inferential meaning)^21^. Given the very rapid and distributed nature of these processes^22,23^, previous research into lexical access has been unable to disentangle retrieval from related computations, such as inferring semantic coherence. Much work into linguistic coherence has utilized scalp event-related potentials or functional MRI (fMRI)^24,25^, which lack the fine spatiotemporal resolution needed to comprehensively map cortical responses. Under some analyses, the cortical substrates for language and semantic processing overlap^26,27^, while others point to dissociability^28^. Across inferior frontal, inferior parietal, and posterior temporal cortices, it remains unclear if there are functionally specialized subregions for diverse semantic processes^29–31^.

In order to isolate sites involved in distinct semantic processes we used an orthographic sentence comprehension and linguistic reference paradigm in a large cohort of subjects undergoing intracranial electrocorticography for the evaluation of medically refractory epilepsy (iEEG) (58 patients; 11,328 electrodes)^32,33^. We used broadband high gamma activity (BGA; 70–150 Hz), which strongly correlates with the fMRI blood oxygen level-dependent (BOLD) signal, to index local cortical processing^34^. We comprehensively mapped BGA responses across the language-dominant cortex by presenting sentences triggering inferential semantics (as opposed to directly showing a picture of an object) that were either referential^35^ or non-referential to common objects^36^.

Patients generated common object names in response to written descriptions of variable lengths via rapid serial visual presentation (500 ms per word). Our orthographic stimuli afforded temporally precise lexical inputs and minimize integrative processes that are intrinsic to continuous auditory or visual inputs. The final word dictated whether the sentence referred to a common lexical item, manipulating the *referential* nature of the description. Within non-referential trials, we manipulated their semantic *coherence*. This allowed us to isolate cortical dynamics of semantic convergence to a common lexical item. To illustrate these concepts, the sentences “The part of the tree that grows underground” and “A person at the circus who makes you laugh” permit successful lexical access (e.g., referring to *roots* and *clown*). However, “A person at the circus who makes you commute” does not permit lexical access to a common name. Yet, this sentence retains semantic coherence and contains what we will call a *weak violation* of lexico-semantic rules (i.e., it is conceptually possible that there are people at circuses who make other people commute, but there is no common word for this concept). In contrast, “A place where oceans shop” is non-referential to a common word but is also semantically incoherent, and represents a *strong violation* (see Methods). For all Non-Referential trials, patients were instructed to verbally respond “nonsense”. As such, our notion of referential meaning conforms to a broader sense commonly used in the psycholinguistics literature pertaining to inferential semantics and lexical reference, as opposed to a narrower sense pertaining to definiteness and grammatical specificity regulated by functional syntactic structure.

Lastly, we collected norming data (*n* = 80) to quantify the point of what we term semantic ‘narrowing’ that enabled an analysis of the timing and extent of the lexicon search effort for all Referential sentences, e.g., “It’s white and falls from the sky in winter” was likely to be inferred as *snow* before the final word, while other trials did not enable inference until the final word, e.g., “An object used for weighing”. This pertains to the notion of cloze probability or the extent to which a particular item is predicted from a context, and likely indexes composition-driven lexical search effort and hence allows us to explore an additional feature of lexical access^37–39^.

**Fig. 1:**
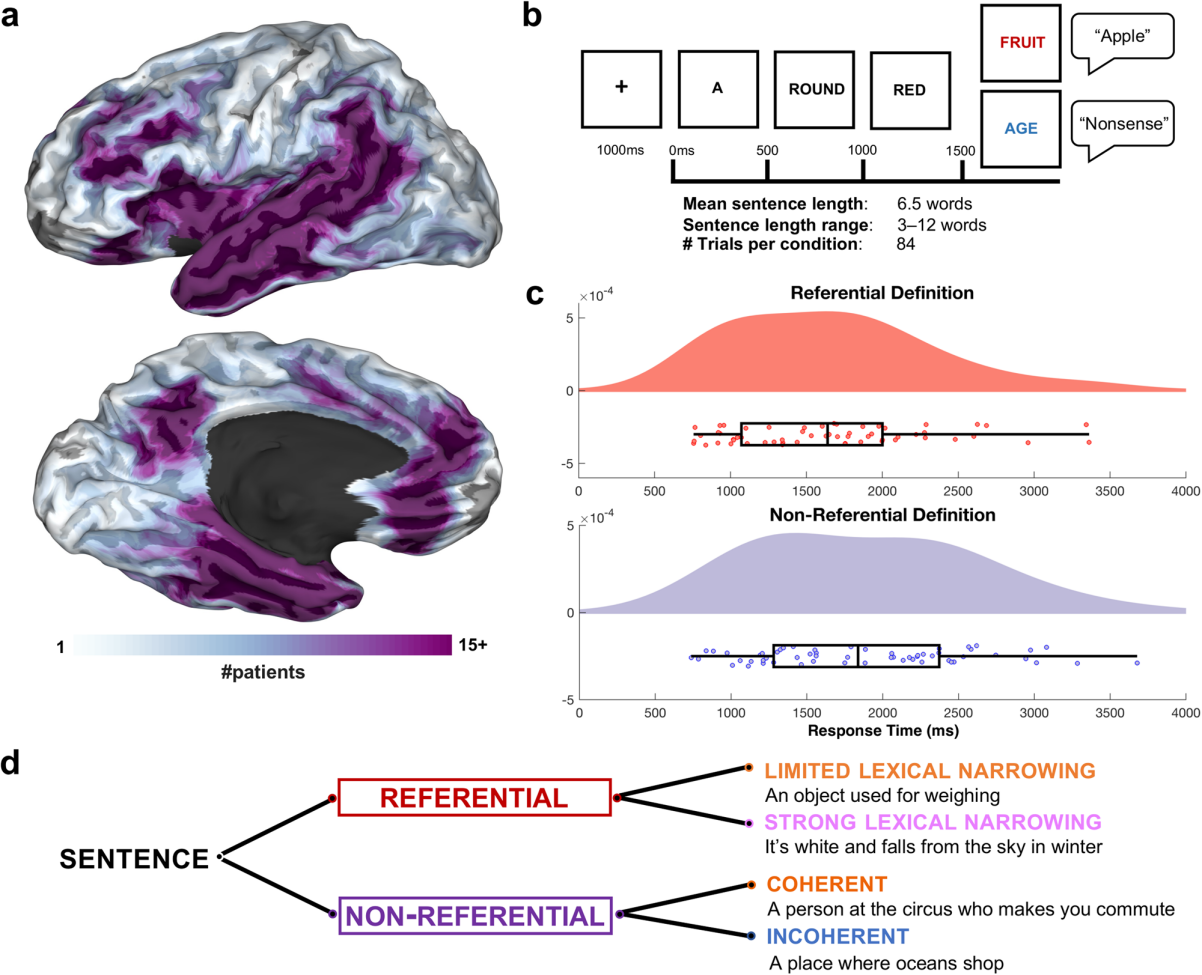
Intracranial electrode coverage with experimental paradigm. **a** Group electrode coverage map, plotted on a semi-inflated standardized N27 surface. **b** Experimental design. Sentences were presented orthographically in rapid serial visual presentation, 500 ms per word. Patients verbally articulated their responses. **c** Time from the offset of the final word to the onset of patient verbal articulation. *N* = 58 patients for each plot (Referential and Non-Referential), the total number of patients and their corresponding articulation onset time for both conditions. Minimum (Referential: 757 ms; Non-Referential: 737 ms) and maximum (Referential: 3362 ms; Non-Referential: 3678 ms) values are plotted alongside the top and bottom 50% of values, centered around the means. Source data are provided as a Source Data file. **d** Sentence conditions split by referential status, with example trials. The two main semantic processes derived here are reference and coherence, with narrowing modulations exposing the degree of lexical search effort involved in reference resolution.

To summarize our conditions, on the one hand, we have integrative lexical access (‘Reference’) and a measure of how effortful this is (‘Narrowing’), and on the other hand, we have semantic coherence (splitting up Non-Referential trials into coherent vs. incoherent). Our design hence afforded four main experimental conditions, focusing primarily on the effects of semantic reference and coherence (Fig. 1d) (Non-Referential: coherent vs. incoherent; Referential: strong narrowing vs. limited narrowing). These two semantic processes are likely to be somewhat correlated but expected to be engaged to varying degrees during sentence comprehension, depending on the particular task and linguistic content. It is an open question whether these processes engage overlapping or non-overlapping cortex at parallel or distinct time intervals. Together, these analyses enabled us to comprehensively elaborate the spatiotemporal dynamics of semantic integration.

## Results

### Behavioral performance

Individual reaction time (Fig. 1) averaged 1765 ms (SD: 680 ms) after the offset of the final word in the sentence. Referential trials had significantly faster articulation reaction times than non-referential trials (paired *t*-test; *t*(1,83): 1.86, *p*-value: 0.032).

### Spatiotemporal dynamics of orthographic sentence processing

**Fig. 2:**
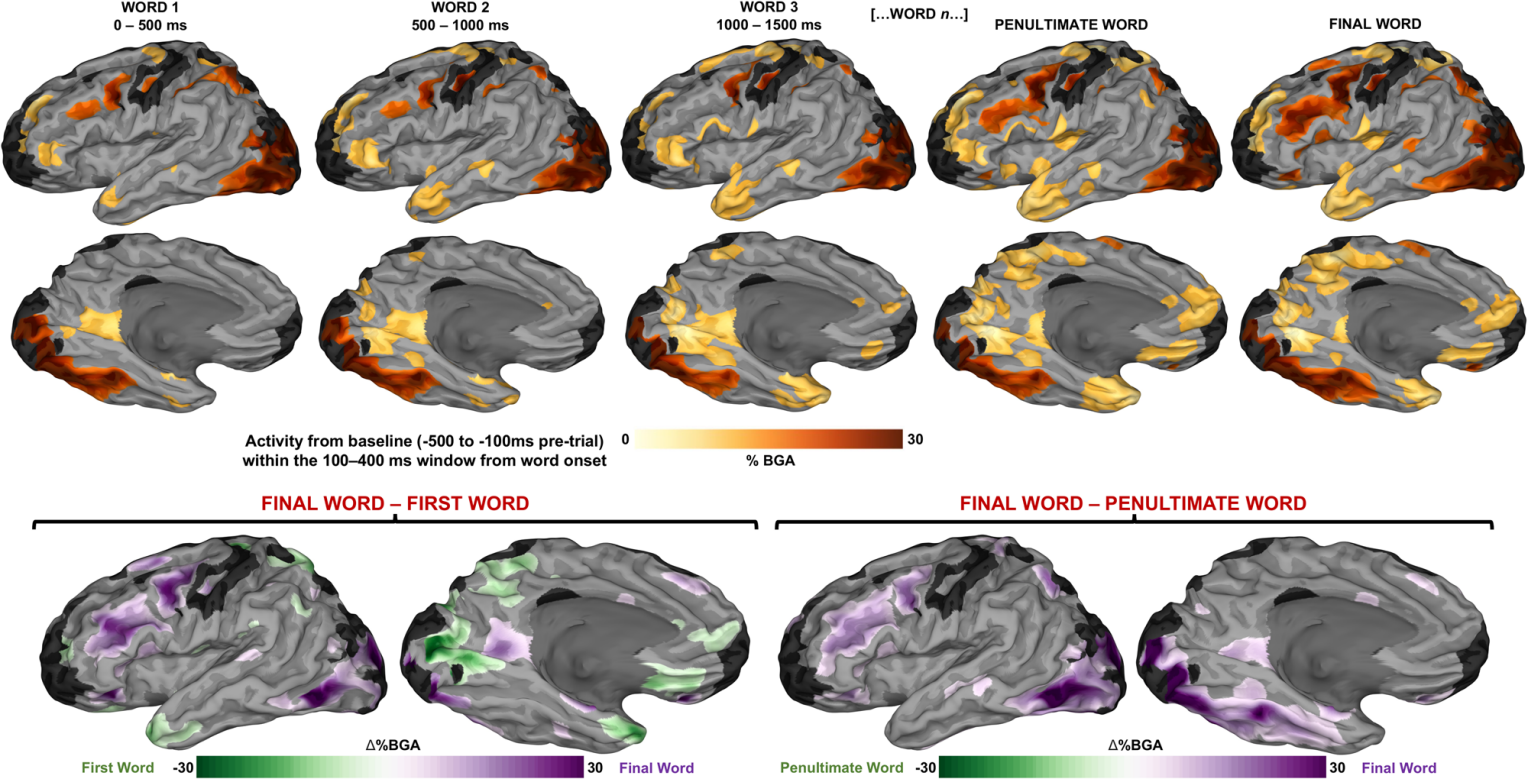
High gamma signatures of orthographic sentence processing. Responses to orthographic stimuli (averaged across the 100–400 ms window after the presentation of each word), relative to baseline (−500 ms to −100 ms before first word onset) represented using a surface-based mixed effects multi-level analysis (SB-MEMA) (thresholded at *t* > 1.96, patient coverage ≥3, *p* < 0.01). Trials with only three words (2 trials) were excluded from Word 3 maps. Bottom: Contrast for final vs. first word (*left*) and final vs. penultimate word (*right*) across the same window and baseline (thresholded at *t* > 2.57, patient coverage ≥3, corrected *p* < 0.01). One-sided tests were conducted for these contrast SB-MEMAs with a familywise error rate correction and an alpha-level of .01 to correct for multiple comparisons.

To probe the build-up of local cortical activity across successive words, we generated a population-level map of cortical activity using a surface-based mixed-effects multi-level analysis (SB-MEMA)^34,40–42^. This revealed serially increasing activation across the sentence duration in a distributed orthographic sentence processing network (Fig. 2, top). Early activation was found in the inferior frontal gyrus (IFG), medial parietal cortex (MPC), anterior temporal lobe (ATL), and posterior middle temporal gyrus (pMTG), and this was followed by late activation in the ventromedial prefrontal cortex (vmPFC), posterior cingulate and orbitofrontal cortex (OFC). Three regions – the ventral temporal cortex, the inferior lateral temporo-occipital cortex, and the inferior frontal sulcus (IFS) in its entire antero-posterior extent – were active throughout sentence reading and showed a clear increase in BGA at the final word and a broader spread of activity across the sentence (Fig. 2, bottom). Contrasts with the final word against both the first word and the penultimate word further highlight regions that exhibited a relative increase over the course of the trial (Fig. 2, bottom). Non-Referential trials were excluded from analyses involving the final word.

### Reference to common objects

**Fig. 3:**
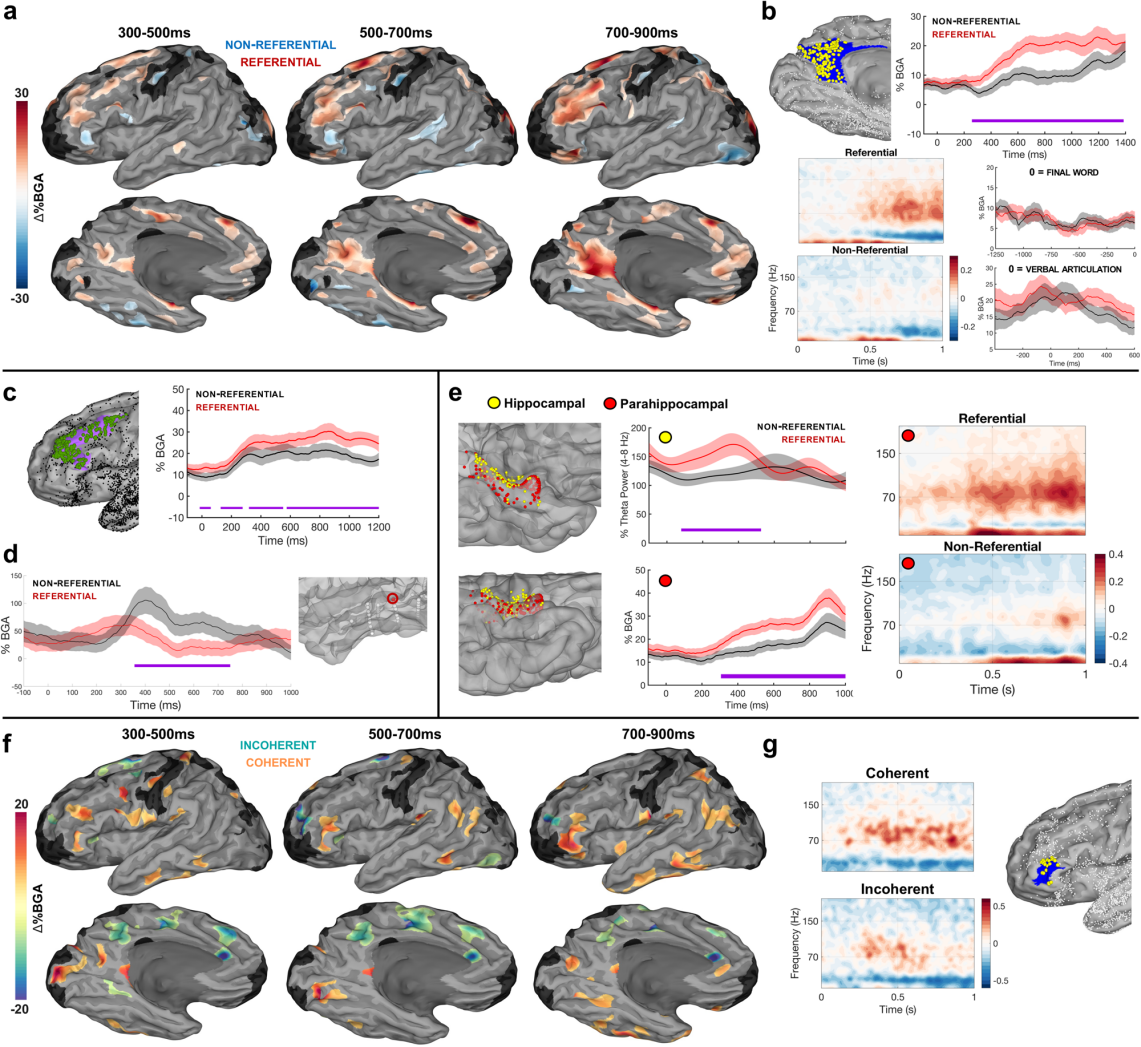
Cortical activity profiles for linguistic reference and coherence. **a** SB-MEMAs for referential vs non-referential trials, with red indexing greater BGA (70–150 Hz) for referential sentences and blue for non-referential sentences (threshold: %BGA > 5%, *t* > 1.96, patient coverage ≥3, *p* < 0.01 corrected). Time 0 ms = onset of the final word. **b** Top Left: Electrodes (yellow dots; *n* = 245) placed within the medial parietal cortex (MPC); other electrodes are small dots. Top Right: Grouped electrode traces for BGA (70–150 Hz) (red: referential; black: non-referential), error bars (colored shading) set at one standard error; significance (FDR-corrected) bars in purple. Time series average of group estimates of BGA percent change ± 1 SEM following final word onset. Bottom Left: Spectrographic representation of grouped electrodes in MPC time-locked to final word onset. Bottom Right: Grouped traces time-locked to pre-final word period (top) and patient verbal articulation (bottom) for MPC. **c** Grouped traces for middle frontal cortex (electrodes: 194), error bars (colored shading) set at one standard error. **d** Single electrode trace from a patient with recordings from posterior superior temporal regions, exhibiting greater BGA for Non-Referential trials, error bars (colored shading) set at one standard error. **e** Left: Recording sites (334 electrodes in 42 patients) placed within either the hippocampus (yellow) or parahippocampal gyrus (red) represented on a standardized pial N27 surface. Middle: Grouped representation of hippocampal theta power (top) and parahippocampal BGA (bottom) time-locked to final word onset, error bars (colored shading) set at one standard error. Right: Spectrographic representation of grouped electrodes for all active electrodes in the parahippocampal cortex. **f** SB-MEMAs for non-referential coherent vs. incoherent trials, with dark orange coloration indexing greater BGA for coherent non-referential sentences and turquoise coloration for incoherent non-referential sentences (same thresholds as (A)). **g** Electrodes and spectrograms for anterior IFG (HCP index: p47r; electrodes: 24; patients: 10) for coherent and incoherent trials.

A comparison of referential vs. non-referential trials at the onset of the final word revealed greater BGA for referential trials in MFG and middle IFS (500–700 ms: *β* = 0.08 (SD: 0.03); *p* < 0.001; 700–900 ms: *β* = 0.10 (SD: 0.05); *p* < 0.001), MPC and parahippocampal cortex (500–700 ms: *β* = 0.11 (SD: 0.06); *p* < 0.001; 700–900 ms: *β* = 0.13 (SD: 0.08); *p* < 0.001), vmPFC (500–700 ms: *β* = 0.07 (SD: 0.01); *p* < 0.001; 700–900 ms: *β* = 0.08 (SD: 0.04); *p* < 0.001), and OFC (500–700 ms: *β* = 0.09 (SD: 0.04); *p* < 0.001; 700–900 ms: *β* = 0.11 (SD: 0.05); *p* < 0.001) (Fig. 3, Supplementary Figs. 1, 2, 4). Non-referential trials exhibited BGA increases relative to referential trials in posterior superior temporal cortex (500–700 ms: *β* = 0.07 (SD: 0.01); *p* < 0.001) and anterior inferior frontal gyrus (aIFG) (500–700 ms: *β* = 0.08 (SD: 0.03); *p* < 0.001) (Fig. 3). These effects were specific to the period immediately after final word onset, as opposed to patient verbal articulation (Fig. 3b). Frontal (Fig. 3c) and medial temporal (Fig. 3e) effects of reference, and an exemplar posterior temporal electrode displaying this non-referential sensitivity (Fig. 3d), are plotted. Across the non-dominant right hemisphere, we also saw general increases for non-referential trials relative to referential trials (Supplementary Fig. 3), although given our significantly reduced electrode coverage over the right hemisphere we refrain from any further observations.

### Semantic coherence

Focusing on non-referential sentences, a whole brain analysis contrasting coherent and incoherent trials showed increased activity for incoherent non-referential sentences in the medial frontal cortex (300–500 ms after the onset of the final word: *β* = 0.10 (SD: 0.04); *p* = 0.001) and superior medial parietal cortex (300–500 ms: *β* = 0.09 (SD: 0.03); *p* < 0.001). The supero-medial parietal activations were seen in loci distinct from with the sites sensitive to referential vs. non-referential contrasts (Fig. 3f). Coherent non-referential sentences resulted in greater BGA in IFS (300–500 ms: *β* = 0.08 (SD: 0.03); *p* = 0.001), aIFG (700–900 ms: *β* = 0.11 (SD: 0.05); *p* = 0.001) (Fig. 3G), angular gyrus (700–900 ms: *β* = 0.06 (SD: 0.01); *p* < 0.001), pMTG (700–900 ms: *β* = 0.08 (SD: 0.03); *p* = 0.002) and OFC (700–900 ms: *β* = 0.07 (SD: 0.01); *p* = 0.001).

### Integrative lexical access

To calibrate our stimuli, we conducted a norming study with native English speakers (*n* = 80) enabling the derivation of the point of what we call *semantic narrowing*, elaborating further on the above process of reference resolution and lexical access. This denotes the probability that the defined object could be identified before the presentation of the final word. Certain sentences yielded probable answers before the final word due to the presence of semantically salient information (e.g., “Something you use to *unlock* a door”), whereas other sentences were ambiguous until the final word (e.g., “What you use to measure *temperature*”). We then contrasted trials that exhibited limited semantic narrowing (i.e., they provided a smaller set of possible answers to the definition before the final word) at the point of final word onset with those exhibiting strong narrowing (Supplementary Fig. 5). This was the most conservative means of isolating a contrast between limited and strong narrowing due to variability in the position of narrowing words throughout the sentence, as well as our inability to ensure that patients were specifically constraining their definitional search at time points concurrent with the mid-sentence positions in the norming data. Articulation reaction times (Supplementary Fig. 5A) did not differ between narrowing conditions (strong narrowing: 1886 ± 708 ms; limited narrowing: 1904 ± 699 ms; paired *t*-test, *t*(1,34) = −0.09, *p* = 0.46), which possibly suggests distinct mechanisms from those implicated in cloze tasks^38^. There was no significant difference between conditions with respect to sentence length (strong narrowing length; M: 6.8; range: 5 (4–9); limited narrowing length; M: 6.4; range: 5 (3–8); paired *t*-test, *t*(1,41) = 1.36, *p* = 0.08). There was also no significant difference in the frequency of the final word between the two conditions (strong narrowing final word frequency, SUBTLEXus log-frequency (Lg10CD) M: 3.09; range: 2.81 (1.11–3.92); limited narrowing final word M: 2.90; range: 2.7 (1.17–3.91); paired *t*-test, *t*-(1,41) = −1.2; *p* = 0.240), and the cortical loci we document below are not typically implicated in lexical frequency sensitivity^43^.

Referential trials with limited semantic narrowing revealed greater BGA than strong narrowing trials in and around posterior superior temporal sulcus (pSTS) beginning approximately 250 ms after the onset of the final word and lasting until approximately 900 ms (300–500 ms: *β* = 0.10 (SD: 0.04); *p* = 0.001; 500–700 ms: *β* = 0.08 (SD: 0.03); *p* < 0.001) (Supplementary Fig. 5b). Greater BGA for limited narrowing trials was also found in MPC (500–700 ms: *β* = 0.12 (SD: 0.07); *p* = 0.003), IFS (300–500 ms: *β* = 0.11 (SD: 0.05); *p* = 0.008; 500–700 ms: *β* = 0.12 (SD: 0.07); *p* = 0.004), anterior temporal lobe (500–700 ms: *β* = 0.06 (SD: 0.01); *p* = 0.004) and OFC (500–700 ms: *β* = 0.12 (SD: 0.07); *p* < 0.001). Spectrograms depicting effects in IFS and pSTS are plotted (Supplementary Fig. 5C), alongside exemplar electrodes from pSTS (Supplementary Fig. 5D).

## Discussion

**Fig. 4:**
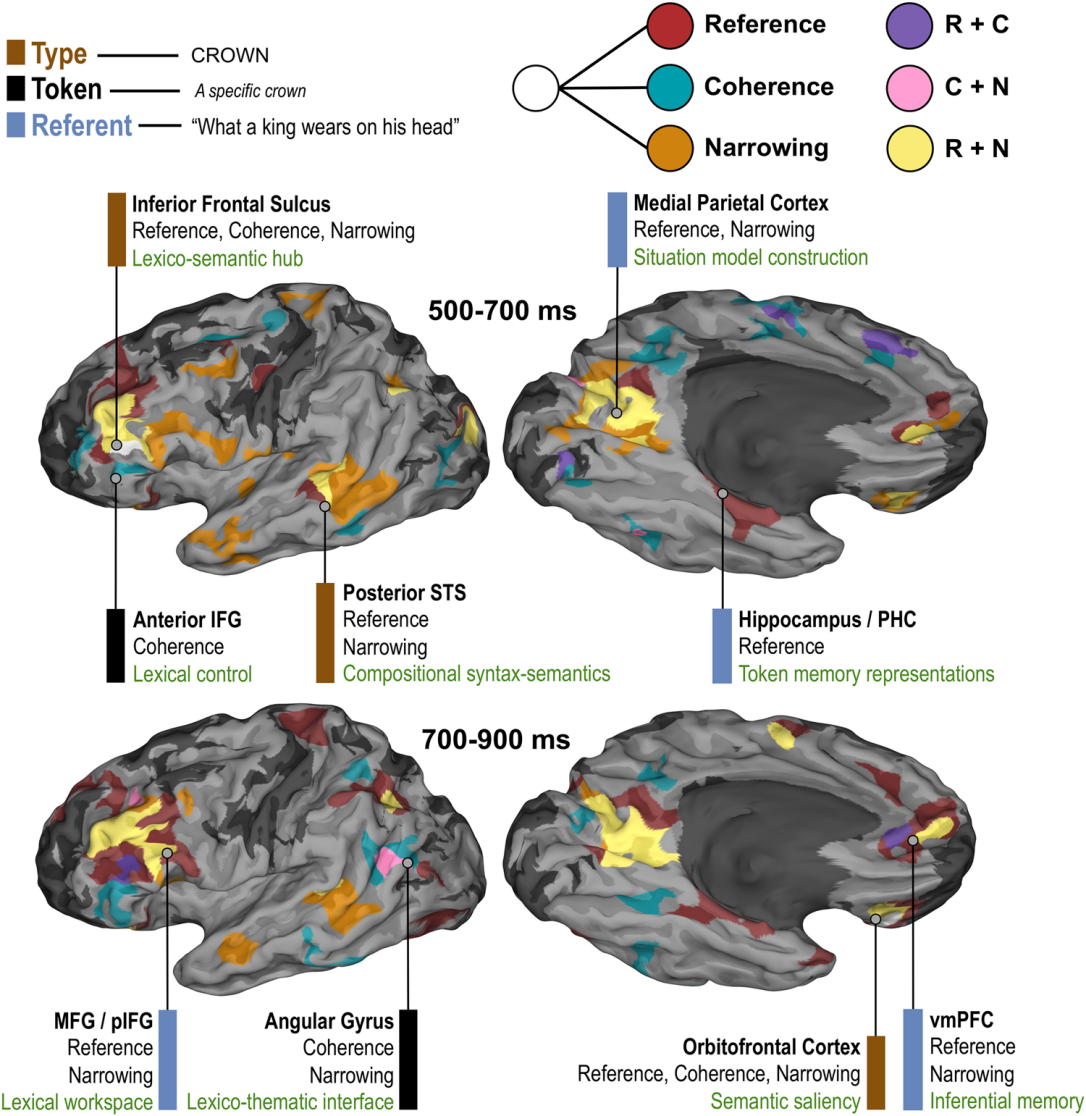
Summary model for distinct components of linguistic meaning derived from a conjunction SB-MEMA. Model derived from present analyses of reference resolution (referential vs. non-referential sentence endings) and its difficulty (limited vs. strong narrowing), and semantic coherence (coherent vs. incoherent non-referential sentences). Included is the color scheme for Type, Token, and Referent matched to colored bars next to regional descriptions, and also for regions showing some conditional difference for Reference, Coherence, and Narrowing (matched to colored clusters on brain plots) and for combinations of these (e.g., R + C = Reference and Coherence, but not Narrowing). Conjunction SB-MEMAs are plotted depicting regions sensitive to conditional contrasts, regardless of directionality (threshold: %BGA > 5%, *t* > 2.57, patient coverage ≥ 2), across two main time windows of interest (500–700 ms, 700–900 ms). Functional descriptions in green (bottom entries) are inferred from existing literature and are not intended to be monolithic, but specific to the present task context of naming-to-definition (see Discussion).

Our evaluation of the spatiotemporal dynamics of sentence reading identified distinct semantic roles for closely adjacent loci in the lateral inferior frontal and posterior temporal cortex. We examined the effects of integrative lexical access and semantic coherence. Whilst frontal gyral structures are commonly implicated in the literature on linguistic semantics^2,15^, we discovered that the inferior frontal sulcus (IFS) exhibited greater high gamma activity for all semantic contrasts, exhibiting a clear mosaic-like patchwork of activity across its sub-regions (Fig. 4). Although many models have proposed anterior-posterior distinctions for inferior frontal regions with respect to certain higher-order language functions^15,44–46^, our recordings of distinct semantic effects provide a more complex picture. Though we indeed find support for separable semantic effects in anterior and posterior inferior frontal gyrus (IFG) – coherence for the former, and difficulty of integrative lexical access for the latter – there was also found to be some functional overlap (Fig. 4). Neighboring sulcal loci also showed effects that were not only earlier than those in IFG, but also more topographically complex. Centered around IFS and spreading out dorsally towards the middle frontal gyrus and ventrally towards the anterior/posterior inferior frontal gyrus, we uncovered a cortical mosaic of functional sensitivity to reference and coherence, in addition to finding effects of reference resolution difficulty (‘narrowing’). Early processing windows implicated IFS in all aspects of semantics, and in later windows sub-portions of IFS exposed a clearer functional tessellation with some remaining overlap. The orbitofrontal cortex (OFC) was also sensitive to all semantic processes, albeit during a later time window. OFC also became significantly active later in sentence reading, whereas IFS was active at all periods (Fig. 2).

We additionally discovered that the posterior superior temporal sulcus (pSTS) and posterior middle temporal gyrus (pMTG) jointly contributed to all semantic processes. The temporal progression of sensitivity in the posterior temporal cortex had a clear delineation between early effects of reference (in particular, difficult reference via ‘limited narrowing’), and finally coherence. Portions of frontotemporal cortex were engaged for all aspects of linguistic semantics but became rapidly dedicated to certain processes at distinct times. Previous intracranial work has implicated pSTS in the initial construction of meaningful phrases, and IFS in the later evaluation of these phrases^3^. Our results further highlight the joint role of pSTS-IFS in semantic composition demands, with early semantic narrowing effects in pSTS (onset ~250 ms) being followed by effects for all conditions in IFS (~500 ms) (Fig. 4). The temporal progression of early high gamma increases for non-referential over referential items in pSTS, to frontal effects of semantic coherence, is concordant with MEG studies showing the spread of sentence-closure N400 congruity signatures from STS to frontal cortex^47^. Alongside these sites, we found that medial parietal cortex (MPC), hippocampus, parahippocampal gyrus (PHG), OFC, intraparietal sulcus, and ventromedial prefrontal cortex (vmPFC) are engaged during the processing of sentences that permitted inference to common object names.

The network typically implicated in the ability to successfully generate names of visually presented common objects consists of the inferior frontal cortex, mid-fusiform cortex, and the supplementary motor area^48–51^. Regions discovered from fMRI that are involved in lexical access for both picture-derived and sentence-derived access are mid-fusiform cortex, inferior frontal gyrus, and middle temporal gyrus^52^. Our results provide additional details about particular integrative components of semantic processing, with our analyses dissociating lexical search difficulty (within referential trials) and semantic coherence (within non-referential trials). We also reported on the basic build-up of local activity across normal sentence processing (Fig. 2), exposing regions more likely to be involved in lexical access from the Final Word – Word 1 contrast, and regions more likely to be involved in semantic integration from the Final Word – Penultimate Word contrast. We assume that the major nodes we identified in this network are responsible for distinct components of semantic integration, with areas such as pMTG, posterior cingulate, OFC, and vmPFC becoming significantly active only later in the sentence. As such, processes relating to situation model construction may be subserved by these regions, with vmPFC being implicated recently in associative inference and memory integration^53^. We further discuss below the likely contributions of regions implicated in our results to orthographic sentence processing in the service of lexical access (Fig. 4).

Previous research into linguistic reference to both visual objects and sounds implicated greater engagement of MPC from 500–600 ms after the orthographic presentation of the referential word in a sentence^54^. These regions form part of the default network, which is engaged during endogenous attentional tasks (i.e., directing attention at ‘internal’ processes and events, in contrast to ‘external’ data) and interoceptive monitoring, with representational search likely being greater for sentences permitting successful selection of objects from the lexicon^55^. Retrosplenial cortex forms a major part of this MPC response. This region encodes (i) predictions and error corrections for current sensory states with internal representations of the environment, and (ii) shifting perspectives and mental reference frames, both of which support endogenous attention and likely facilitate task-related representational search^56^. The clear involvement of MPC across a range of semantic integration processes may relate to the recently documented human-specific sulcal morphology of posteromedial cortex^57^. Default network activity is related to processing rich representations of events, either real or imagined, of the kind inferred by patients in our task. Relatedly, our effects in dorsal frontal sites seem to overlap with a node in the multiple demand network^58,59^, which has been implicated in linguistic processing difficulty, seemingly congruent with our semantic narrowing effects in IFS and MFG.

Linguistic reference to coherent entities has been shown to implicate medial frontal and medial parietal regions, along with perisylvian language regions^19^. Sentence pairs introducing a conjoined subject (e.g., “John and Mary”) result in greater MPC engagement compared to individual referents^60^. Referentially ambiguous pronouns (e.g., “Saul told Mike that he…”) have been shown to modulate medial parietal activity^18^. Our findings concerning medial parietal regions seem in accord with the involvement of the MPC in the posterior medial system^61^, which is part of the distributed memory network and is involved in context memory, constructing situation models, and combining concepts from distinct categorical domains^62–66^. The MPC has been shown to contain selective regions for the recall of information about familiar people and places but does not demonstrate activity during visual object naming^67,68^, and the precuneus has been implicated in the encoding of complex memories and actions^69^. Our results indicate involvement not just in the above forms of grammatical co-reference, but also in inferential semantics pertaining to basic lexical access.

In addition to MPC, the posterior medial system incorporates the hippocampus and PHG. Hippocampal theta tracks the amount of contextual linguistic information in a sentence, such that in predictable sentence contexts theta power increases during sentence presentation^70^. Other recent intracranial work has indicated a role for the medial temporal lobe in verbal working memory^71^. This suggests that portions of the hippocampus and PHG actively relate incoming words to sentence contexts and contribute memory-related representations needed to successfully search the (narrowed) lexicon. We discovered early hippocampal theta power increases for referential trials (100–400 ms) followed directly by BGA increases in PHG (300–1000 ms); it seems plausible that joint MPC-hippocampal engagement underlies contextual associations^72–74^.

Our results are also concordant with the findings that hippocampal-complex damage leads to impairments in semantic association tasks testing words learned prior to damage^75^, and that hippocampal theta power increases during episodic memory retrieval^76^. It has been argued that the hippocampus might contribute to language’s facility for displacement^77^, or the capacity to refer to objects/events outside the current spatiotemporal context, and also the tracking of situation/context boundaries^78^, potentially concordant with its present involvement in inferential semantics.

Some of our stimuli that did not refer to a common object were semantically coherent, while others were incoherent (Fig. 1). When contrasting these non-referential trials, we discovered that coherent sentences resulted in greater BGA in aIFG, IFS, angular gyrus, OFC and pMTG relative to incoherent sentences. This effect in aIFG (onset around 300 ms) possibly indexes the entry of the final word into the workspace and the successful wrap-up effect for semantically legal structures, and the subsequent control of the appropriate lexical category^15,79^. Previous work has implicated IFG in various semantic processes during different tasks such as plausibility and acceptability judgements^44,80^. Our results are generally in line with the role of IFG in effortful lexico-semantic processing^81^, but afford a more nuanced picture: Lateral aIFG exhibits greater BGA for non-referential relative to referential sentences, and neighboring aIFG and IFS show greater BGA for semantically coherent non-referential sentences, relative to semantically incoherent non-referential sentences. This leads to a fine-grained spatiotemporal map of anterior IFG activity. Lateral aIFG activity marks referential violations and cortex abutting IFS responds to semantic coherence. We note that during sentence comprehension (Fig. 2) these portions in aIFG and IFS are engaged jointly, suggestive of coordinated semantic processes, dissociable with fine spatiotemporal resolution and appropriate behavioral contrasts. Indeed, there are also a number of semantic integration processes that fall outside of our current focus on reference and coherence^14^, and we expect future intracranial work can help isolate even further the spatiotemporal dynamics of the semantics network.

We also note that inferior parietal regions (angular gyrus) were implicated broadly in semantic coherence, with a smaller region being implicated in both semantic coherence and effortful reference. This region was also only moderately active towards the end of sentence comprehension, as in a previous MEG study of sentence processing (Fig. 1)^82^. Though not as engaged as other regions, these results support suggestions that this region codes for event/thematic structure^15,83^ and may be involved in more incremental aspects of event semantics rather than grammatical processing, thus being recruited for “coherent sentence meaning”, as Matchin and colleagues claim^82^.

Referential trials that exhibited a limited degree of semantic ‘narrowing’ (i.e., that did not clearly reduce the space of possible lexical items to select for articulation) resulted in greater recruitment of IFS, pSTS, MPC, and ATL. Recruitment of pSTS was earliest (approximately 250 ms) and may pertain to composition-related demands or lexical search effort, indexing greater engagement of compositional processing due to greater integrative lexical search demands^15,84^, in particular given previous intracranial work implicating this region in basic semantic composition and lexicality^3^, and suggestions that pSTS acts as an interface between externalization and syntactic/semantic representations^85^. ATL engagement is in line with its apparent role in conceptual processing and entity-related (e.g., common object) representations^86^. Though our two ‘narrowing’ conditions did not strictly isolate the more fundamental processing stage of lexical access (since ‘limited narrowing’ sentences afford varying degrees of lexical access facilitation), we note that future research with other paradigms aimed at definitively disambiguating lexical access from semantic processes is needed to explore this. Certainly, it seems clear from prior lesion studies that patients with chronic left prefrontal cortex damage exhibit impaired lexical selection^76^. We also note that the closely interwoven anatomical localization of the reference and narrowing effects indicates the existence of a ‘core’ integrative lexical access network, flanked by ‘peripheral’ regions that are recruited in cases of greater referential difficulty (i.e., ‘limited narrowing’ trials). For example, consider the organization of effects in posterior temporal, inferior frontal, and medial parietal areas, where regions sensitive to both reference and narrowing are flanked by regions only sensitive to reference and/or narrowing.

We did not attempt here to map out the word-by-word dynamics of easy/difficult reference resolution, and we instead isolated the time window where we could most confidently isolate differences in integrative lexical access. We recognize the limitations of this analysis, and the clear dynamics we depict in our analyses are conservatively interpreted here as pertaining to some aspect of the reference resolution process (i.e., coding for semantically salient information, or some process of set-reduction over the course of the sentence).

Recent intracranial work^37^ reported effects of sensitivity to cloze probability in Broca’s area in six patients during a sentence completion production task. Our results suggest that a smaller region of the frontal cortex (middle IFS) is engaged during lexico-semantic search, not necessarily tied to next-word statistics. In addition, in contrast to Wang and colleagues who reported on a small cohort of patients with subdural arrays, we used a combination of subdural grids and depth electrodes for extensive gyral and sulcal coverage in a large cohort. Other recent intracranial work points towards IFS sensitivity to lexical selectivity during single-word production^87^.

While medial brain structures often get overlooked in models of higher-order language, our results suggest that a more extended network is involved in elementary components of sentence comprehension and lexical access. We provide a general model for the neural representation of linguistic meaning derived from a conjunction map of the above analyses (Fig. 4), highlighting also general engagement in processes pertaining to higher-order semantics and relevant to the present task context. While the annotated descriptions are inferred based on contrasts of interest (see below), the cortical surface maps and their coloration are derived from a conjunction SB-MEMA analysis (see Methods, and figure legend). We plot effects pertaining to the two central psycholinguistic processes explored in this article: integrative lexical access and the derivation of semantic coherence. The effects of reference, and the related measure of narrowing, are plotted alongside the effects of coherence (Fig. 4).

Our results can also be viewed in the context of the three core components of linguistic meaning – Types, Tokens, and Referents. A type is a general category of an entity (e.g., flower), a token is a particular concrete instantiation (e.g., a specific flower), and a referent arises via the explicit denotation of a token (e.g., “That yellow thing in the garden”). Previous work has claimed that these components significantly implicate left temporal (Types), inferior frontal (Tokens), and inferior parietal (Referents) cortices^1^. However, our results motivate some potential revisions to this architecture. Our paradigm involved descriptions of referents calling upon conceptions of general types. However, our paradigm was not designed to specifically adjudicate between these three aspects of meaning, and so our association between semantic components and conditional effects should be taken as purely preliminary and speculative, intended to direct future inquiry and to map out areas of the brain that we can at least *rule in* with respect to semantic components. That is to say, we cannot definitively rule out the involvement of the posterior temporal cortex in, for example, establishing referents; we can only rule it in with respect to being recruited for integrative lexical access. Nevertheless, the immediate compatibility here appears to be between our parietal effects and the connections with processing the discourse referent. MPC is predominantly sensitive to reference and narrowing and may form part of the lexical search process and the construction of Referents. We note that although this region is involved in general category sensitivity (e.g., faces, scenes), strictly within the context of our task its involvement may be centered on establishing discourse referents. Given the role of the (para)hippocampus in episodic memory, the activity differences we found exclusively for the referential/non-referential contrast may index referent-specific memory traces. The involvement of angular gyrus and anterior IFG in coherence points to a sensitivity to specific Tokens, which cannot be generated from non-referential incoherent sentences.

The sensitivity of pSTS mostly to semantic narrowing but also jointly to referential violations, in addition to its previously documented role in basic semantic composition^3^ and its greater engagement for inferential naming over picture naming^21^, points towards an involvement in semantic Types. The clear sensitivity of middle IFS to all semantic processes also points towards an involvement in Types. Both pSTS and IFS are reliably active during semantic unification operations^88^. Lastly, the sensitivity of OFC to all effects, albeit only during late stages (Supplementary Fig. 4), points towards involvement in Types, with this region being commonly implicated in aspects of semantic saliency and control^89,90^. We also note that all three of these regions (pSTS, middle IFS, OFC) were not activated early in the sentence (Fig. 1), only towards the end of the sentence. Indeed, while anterior IFS and posterior IFS were both active from words 1 and 2, there was a notable absence of middle IFS activity during early sentence processing, the locus of our mosaic for semantic integration. This suggests again that the effects documented here pertain to higher-order semantic integration processes relevant to type sensitivity. It may be possible to adjudicate here between lexico-semantic engagement (pSTS, IFS, OFC) and domain-general semantic memory activation (dorsal frontal, medial parietal, medial temporal)^91,92^, in particular, given that PHG, precuneus, and vmPFC are all implicated in general semantic processing^93^.

We again stress here that our experimental paradigm and analyses were optimized to evince the effects of integrative lexical access and semantic coherence. We have speculated some promising and plausible connections to broader concepts in semantics (Fig. 4) as a means of extending the potential scope of how we might use our results to inform more general concerns in the cognitive neuroscience of language.

The Controlled Semantic Cognition account^94,95^ is another candidate for helping to frame our results. Under this account, semantic cognition is split into a *representation* component and a control component, for storing and manipulating semantic information during verbal and non-verbal tasks. Our results implicate many cortical regions involved in representational storage, but also cognitive control regions (i.e., IFS, IFG, pMTG). For example, dorsolateral PFC has shown increased BOLD responses when semantic selection demands are high, whereas ventral PFC and pMTG have shown increased activation during the retrieval of weak semantic associations^94^. Moreover, activation in the intermediate middle-lateral PFC has been found to correlate with *both* of the above demands. This model seems to be sympathetic to our discovery of (i) effects of reference in the middle frontal gyrus and IFS, (ii) effects of semantic coherence in pMTG and anterior IFG, and (iii) a region in middle IFS that responds to all semantic demands. We note that our discovery of a cortical mosaic for semantic structure in IFS is sympathetic to the recently uncovered frontal organization of semantic control, which is “sandwiched” between the multiple-demand and default-mode systems^96^. Our results also help emphasize contributions from medial cortical structures within this Controlled Semantic Cognition framework, such as the MPC.

Overall, despite the limitations of our experimental paradigm, our work suggests that current models apportioning clear frontal, temporal, and parietal separation between semantic integration processes need to accommodate the richly interwoven, mosaic-like architecture of the natural language semantics system^3,97–99^.

We reported extensive whole brain intracranial mapping of semantically coherent orthographic sentence representations, providing insights into semantic integration. We discovered complementary cortical mosaics for semantic integration in the posterior temporal and inferior frontal cortex, recruited for distinct semantic demands. We documented diverse roles for posterior temporal and inferior frontal language regions and dissociated regional contributions to distinct semantic processes. In particular, our intracranial recordings afforded access to sulcal structures as well as the lateral surface of the cortical mantle, with IFS (early sensitivity) and OFC (late sensitivity) uniquely responding to all semantic processes, potentially indexing their roles as higher-order lexico-semantic hubs or sites of computing semantic saliency. Rather than finding posterior temporal and inferior frontal engagement only for successful lexical reference, or only for semantic coherence, we found that partially overlapping sub-regions of these loci are engaged in both (within pSTS, IFS, and aIFG). This topography of functional arrangement implies the existence of small scale regional networks in the lateral frontal and temporal cortex that then connect with the larger scale semantic network distributed across lobes. It remains an open question whether a similar mosaic-like cortical organization underlies the representation of syntactic/grammatical processing and might account for various language dysfunctions^100,101^. A clearer understanding of these systems will pave the way for deeper insight into developmental dyslexia, and acquired language impairment and potentially enable better neuro-rehabilitative and neuroprosthetic approaches for these disorders^102^.

## Methods

### Participants

58 patients (11 male, 18–41 ± 5.7 years, 2 left-handed) participated in the experiment after written informed consent was obtained. All were native English speakers with no history of language deficits. Participants with significant additional neurological history (e.g., previous resections, MR imaging abnormalities such as malformations or hypoplasia, or those with prosopagnosia) were excluded. All patients were subject to full neuropsychological assessments (FSIQ scores: 94.3 ± 13.4). All experimental procedures were reviewed and approved by the Committee for the Protection of Human Subjects (CPHS) of the University of Texas Health Science Center at Houston as Protocol Number: HSC-MS-06-0385. Given that electrode placement in these participants was for clinical need rather than experimental purposes, no statistical methods were used to pre-determine sample sizes, but the number of participants required was based on providing adequate coverage of the language areas being studied.

### Electrode implantation and data recording

Data were acquired from either stereotactically placed depth electrodes (sEEGs; 56 patients) or subdural grid electrodes (SDEs; 2 patients). SDEs were subdural platinum-iridium electrodes embedded in a silicone elastomer sheet (PMT Corporation; top-hat design; 3 mm diameter cortical contact) and were surgically implanted via a craniotomy^34,103–105^. sEEGs were implanted using a Robotic Surgical Assistant (ROSA; Medtech, Montpellier, France)^106,107^. Each sEEG probe (PMT corporation, Chanhassen, Minnesota) was 0.8 mm in diameter and had 8–16 electrode contacts. Each contact was a platinum-iridium cylinder, 2.0 mm in length and separated from the adjacent contact by 1.5–2.43 mm. Each patient had 12–20 such probes implanted (total electrode count: 11,328; M = 199). Following surgical implantation, electrodes were localized by co-registration of pre-operative anatomical 3 T MRI and post-operative CT scans in AFNI^108^. Electrode positions were projected onto a cortical surface model generated in FreeSurfer^109^, and displayed on the cortical surface model for visualization^104^. Intracranial data were collected during research experiments starting on the first day after electrode implantation for sEEGs and two days after implantation for SDEs. Data were digitized at 2 kHz using the NeuroPort recording system (Blackrock Microsystems, Salt Lake City, Utah), imported into Matlab, initially referenced to the white matter channel used as a reference for the clinical acquisition system, and visually inspected for line noise, artifacts, and epileptic activity. Electrodes with excessive line noise or localized to sites of seizure onset were excluded. Each electrode was re-referenced to the common average of the remaining channels. Trials contaminated by inter-ictal epileptic spikes and trials in which participants responded incorrectly were discarded.

### Stimuli and experimental design

Patients were asked to quickly and accurately articulate the names of common objects in response to orthographic descriptions (mean: 6.5 words, range: 3–12 words). Sentences varied in length (3–12 words) to reduce the predictability of the location of the final, definition-determining word. A fixation cross was presented in the center of the screen for 1000 ms, followed by each successive word in the sentence, each appearing for 500 ms, and then a blank screen was presented for 1 s. Patients had 2 seconds to respond. The number of trials per block across the full experiment was as follows: Referential (42), Non-Referential (42). Most patients undertook two blocks (n = 48), with some only completing a single block (*n* = 10). Stimuli were presented using Psychtoolbox^110^ on a 15.4” LCD screen positioned at eye-level, 2–3’ from the patient. Words were presented in sentence case, in Arial font with a height of 150 pixels (~2.2° visual angle). For the incoherent Non-Referential trials, these either involved a clear theta-role violation or a violation of lexical selectional requirements, what we termed a strong violation; e.g., “Something you use to teach a land” (‘land’ cannot be an Agent), “A period of time that lasts seven boats” (‘boats’ cannot be a temporal measurement). In contrast, the coherent Non-Referential trials did not exhibit these specific violations, and only yielded a violation effect pertaining to the non-existence of a specific nominal denoting the inferred concept, what we termed a weak violation (e.g., “A person at the circus who makes you commute” generates a coherent meaning but lacks a corresponding lexical entry satisfying the definitional description). We did not determine differences in vividness ratings for referential vs. non-referential trials, which we recognize could be subject to future research. Our full set of stimuli is available to access via OSF (osf.io/es7y3).

To determine the profile of semantic narrowing effects in our stimuli, we conducted a norming study on a non-clinical population (*n* = 80, 50 female, mean age: 34, range: 18–71). All were native English speakers and had a 90% minimum approval rating on Prolific Academic from which they were recruited to take part in the questionnaire (run via the Qualtrics online platform). Participants were paid 10 USD per hour, and the average completion time was 26 min. Participants were presented with the referential trials from the main experiment, word-by-word, and were asked to type any possible corresponding words that might match the ongoing description upon the presentation of a new word. For example, they were first shown “A”, followed by “A round”, then “A round red”, and finally “A round red fruit”. Most participants answered “apple” after being presented with “A round red”, with many answering “ball” or “circle” after seeing “A round”.

### Signal analysis

A total of 13,298 electrode contacts were implanted in these patients; 9388 of these were included for analysis after excluding channels proximal to the seizure onset zone or exhibiting excessive inter-ictal spikes or line noise. Analyses were performed by first bandpass filtering the raw data of each electrode into broadband gamma activity (BGA; 70–150 Hz) following removal of line noise and its harmonics (zero-phase 2nd order Butterworth band-stop filters)^111^. A frequency domain bandpass Hilbert transform (paired sigmoid flanks with half-width 1.5 Hz) was applied and the analytic amplitude was smoothed (Savitzky-Golay FIR, 3rd order, frame length of 251 ms; Matlab 2019b, Mathworks, Natick, MA). BGA was defined as a percentage change from baseline level; −500 ms to −100 ms before the presentation of the first word in each trial. Periods of significant activation were tested using a one-tailed *t*-test at each time point and were corrected for multiple comparisons with a Benjamini-Hochberg false detection rate (FDR) corrected threshold of *q* < 0.05. For the grouped analysis (across multiple patients), all electrodes were averaged within each subject and then the between-subject averages were used.

To provide statistically robust and topologically precise estimates of BGA, and to account for variations in sampling density, population-level representations were created using surface-based mixed-effects multilevel analysis (SB-MEMA)^34,40–42^. We focus our analyses on the language-dominant left hemisphere (but see also Supplementary Fig. 3 for right hemisphere responses to reference). This method accounts for sparse sampling, outlier inferences, as well as intra- and inter-subject variability to produce population maps of cortical activity. A geodesic Gaussian smoothing filter (3 mm full-width at half-maximum) was applied. The minimum criterion for the family-wise error rate was determined by white-noise clustering analysis (Monte Carlo simulations, 5000 iterations) of data with the same dimension and smoothness as that analyzed. Results were further restricted to regions with at least three patients contributing to coverage. Due to the proximity to loci with typical eye movement artefacts^112^, we also evaluated right-hemispheric ventromedial prefrontal cortex and verified that reported effects were specific to the left hemisphere (Supplementary Figs. 1, 3). Likewise, we confirmed that only the language-dominant left hemisphere medial structures, and not right hemisphere structures, index sensitivity to referential sentences. Lastly, the conjunction SB-MEMA maps (Fig. 4) depict regions sensitive to conditional contrasts, regardless of directionality. The individual MEMA contrast maps were binarized (threshold: %BGA > 5%, *t* > 2.57, patient coverage ≥2) and the logical Boolean values for these three maps were computed to plot which regions showed significant activity differences for each combination (e.g., for narrowing and coherence, but not reference).

Our regions of interest were clustered from a number of Human Connectome Project (HCP) regions^113^: Medial parietal cortex (RSC, POS1, POS2, v23ab, 7 m, 31pv, 31pd, d23ab); hippocampus and parahippocampal gyrus (PreS, EC, PHA1); ventromedial prefrontal cortex (a24, p32, 10r, s32, 10 v, 25, OFC); orbitofrontal cortex (11 l, 13 l); middle frontal gyrus (8 C, 46, p9, 46 v); dorsal inferior frontal gyrus and inferior frontal sulcus (IFSa); posterior superior temporal sulcus (TPOJ1, TPOJ2). For the low frequency analysis in the hippocampus, we manually checked electrode placements to ensure a distinction between electrodes placed within the hippocampus proper and within neighboring parahippocampal sites (Parahippocampus = PreSubiculum/Entorhinal cortex/Parahippocampal Area 1).

The language system is richly interwoven with episodic memory networks, with segregation between these systems resulting more from investigator domain expertise than neurobiological underpinnigs. Given that hippocampal theta is modulated by sentence predictability^70^, theta phase-coupling occurs between the hippocampus and left superior temporal gyrus increases during correct (vs. incorrect) sentences^114^, and theta power in the inferior frontal cortex and central EEG sites is linked to language comprehension^115,116^, we also examined low frequency dynamics across these sites.

Anatomical groups of electrodes were delineated, firstly, through indexing electrodes to the closest node on the standardized cortical surface^117^, and secondly, through grouping channels into parcellations determined by Human Connectome Project (HCP) space^113^. Parametric statistics were used since HCP regions of interest contained >30 electrodes. To determine significant activity increases from baseline, a two-sided paired t-test was evaluated at each time point for each region and significance levels were computed at a corrected alpha-level of 0.05 using an FDR correction for multiple comparisons. To determine a significant difference between conditions in the activation of a region, the cumulative BGA in a specified time window was evaluated with a two-sided paired *t*-test at an alpha-level of 0.01.

### Reporting summary

Further information on research design is available in the Nature Portfolio Reporting Summary linked to this article.

### Supplementary information

Supplementary Information

Reporting Summary

### Source data

Source Data

## Data Availability

The datasets generated from this research are not publicly available due to them containing information non-compliant with HIPAA and the human participants the data were collected from have not consented to their public release. However, they are available on request from the corresponding author. Our full set of stimuli is available to access (osf.io/es7y3). Source data are provided with this paper.

## Code availability

The custom code that supports the findings of this study is available from the corresponding author on request.
