# Supplementary material for: The spatiotemporal dynamics of semantic integration in the human brain
Source: Nat Commun. 2023 Oct 24;14:6336. doi: 10.1038/s41467-023-42087-8 (PMC10598228; doi:10.1038/s41467-023-42087-8)
Supplement: Supplementary file 1 — Supplementary Information [file 41467_2023_42087_MOESM1_ESM.pdf]

The spatiotemporal dynamics of semantic integration in the human brain

Supplementary Information

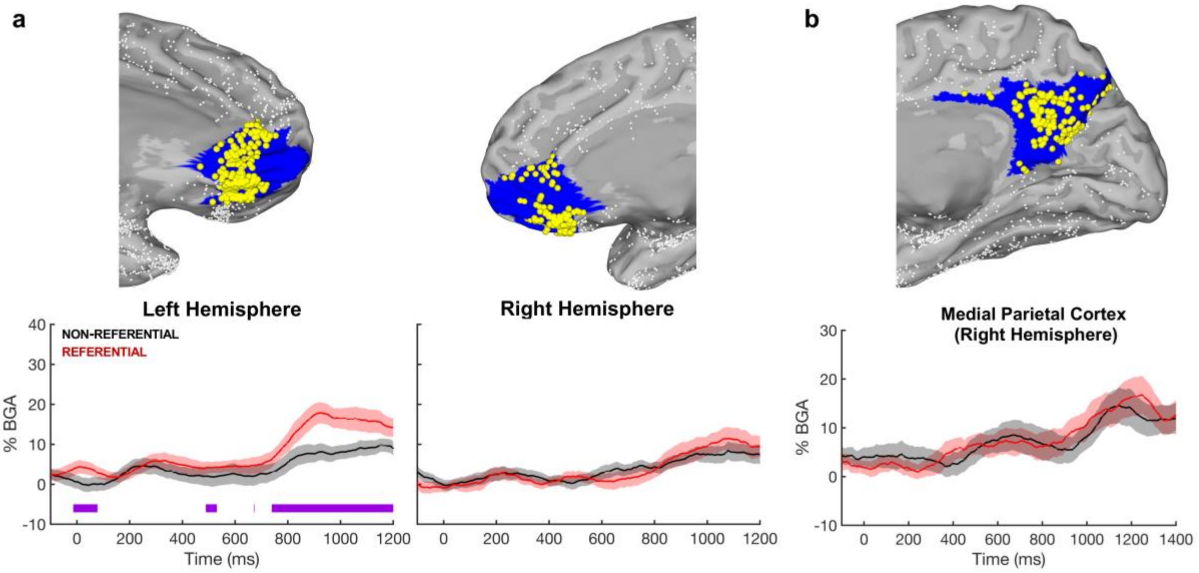

Supplementary Figure 1. **Hemispheric contrasts of reference. a** Electrodes placed within left vmPFC (233) and their corresponding BGA trace, alongside the right hemisphere (126). FDR-corrected significance bars plotted in purple. Time-locked to final word onset. **b** Electrodes placed within the right medial parietal cortex (139) from patients with bilateral coverage (31) and their corresponding BGA traces (for left hemisphere, see Figure 3).

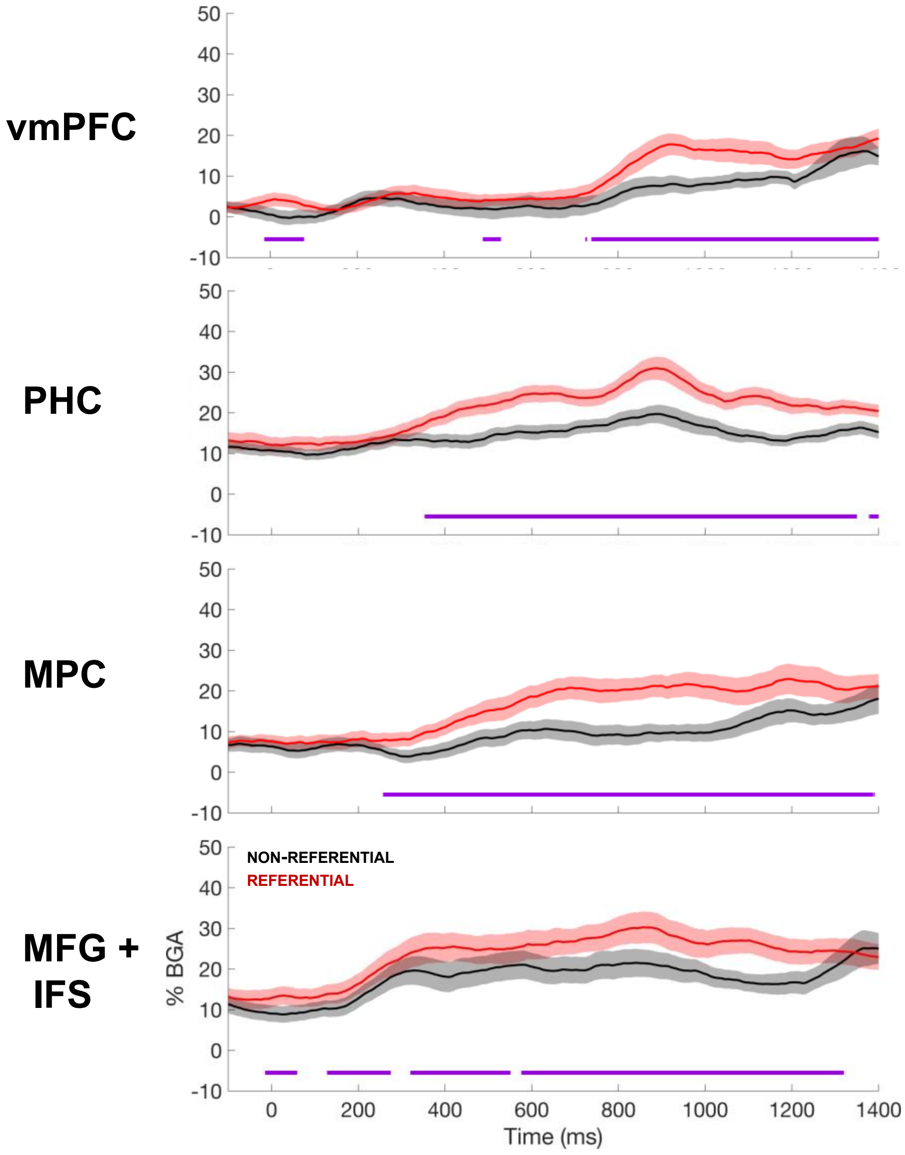

Supplementary Figure 2. **Time course of referential sensitivity.** Major regions of interest and their grouped time traces for referential sensitivity, organized bottom-to-top via earliest to latest effect. MFG (middle frontal gyrus), IFS (inferior frontal sulcus), MPC (medial parietal cortex), PHC (parahippocampal cortex), vmPFC (ventromedial prefrontal cortex).

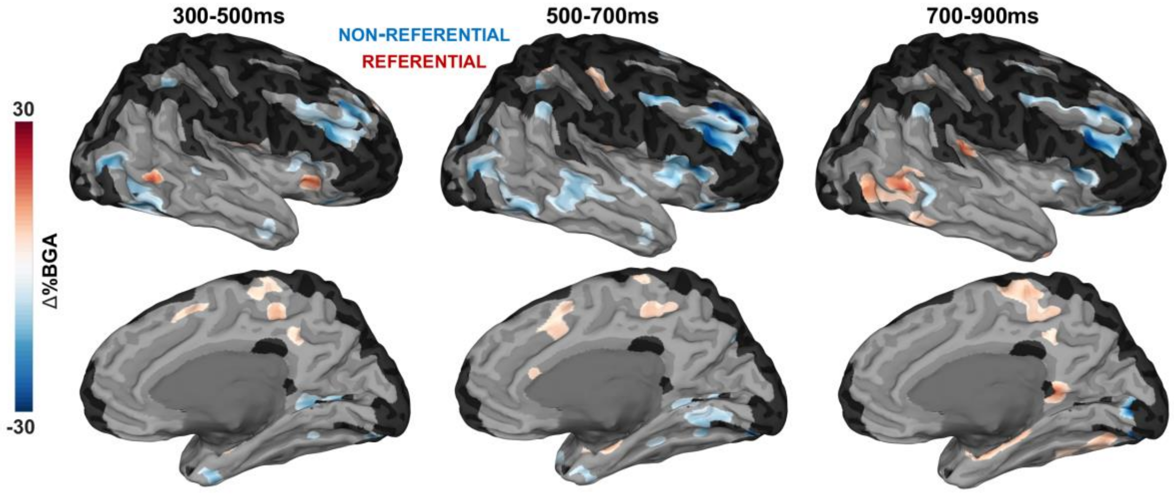

Supplementary Figure 3. **Cortical activity profile for linguistic reference across the right hemisphere.** SB-MEMAs for referential vs non-referential trials across the right hemisphere (non-language-dominant), with red indexing greater BGA (70–150Hz) for referential sentences and blue for non-referential sentences (threshold: %BGA > 5%, t > 1.96, patient coverage ≥ 3, $p < 0.01$ corrected). Time 0 ms = onset of final word.

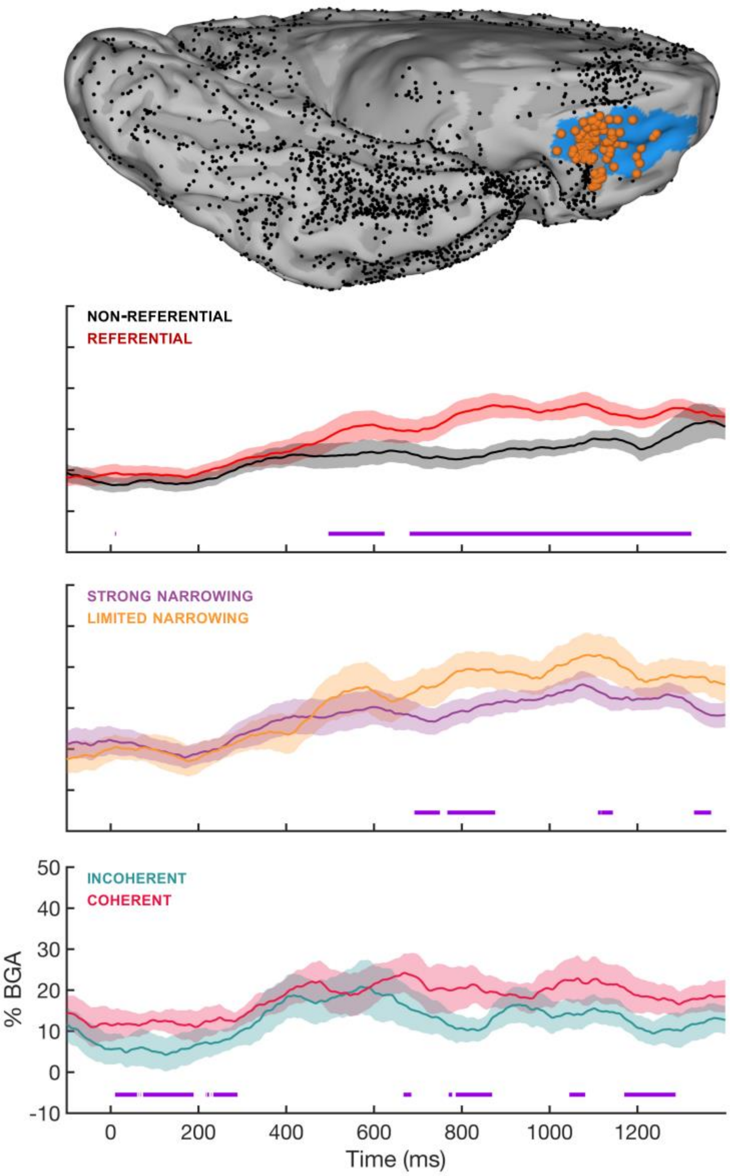

Supplementary Figure 4. **Time course for semantic integration effects in ventral orbitofrontal cortex.** Electrodes (97, across 27 patients) placed within ventral orbitofrontal cortex (HCP index: 11l, 13l) (Top), and time course of effects (Bottom).

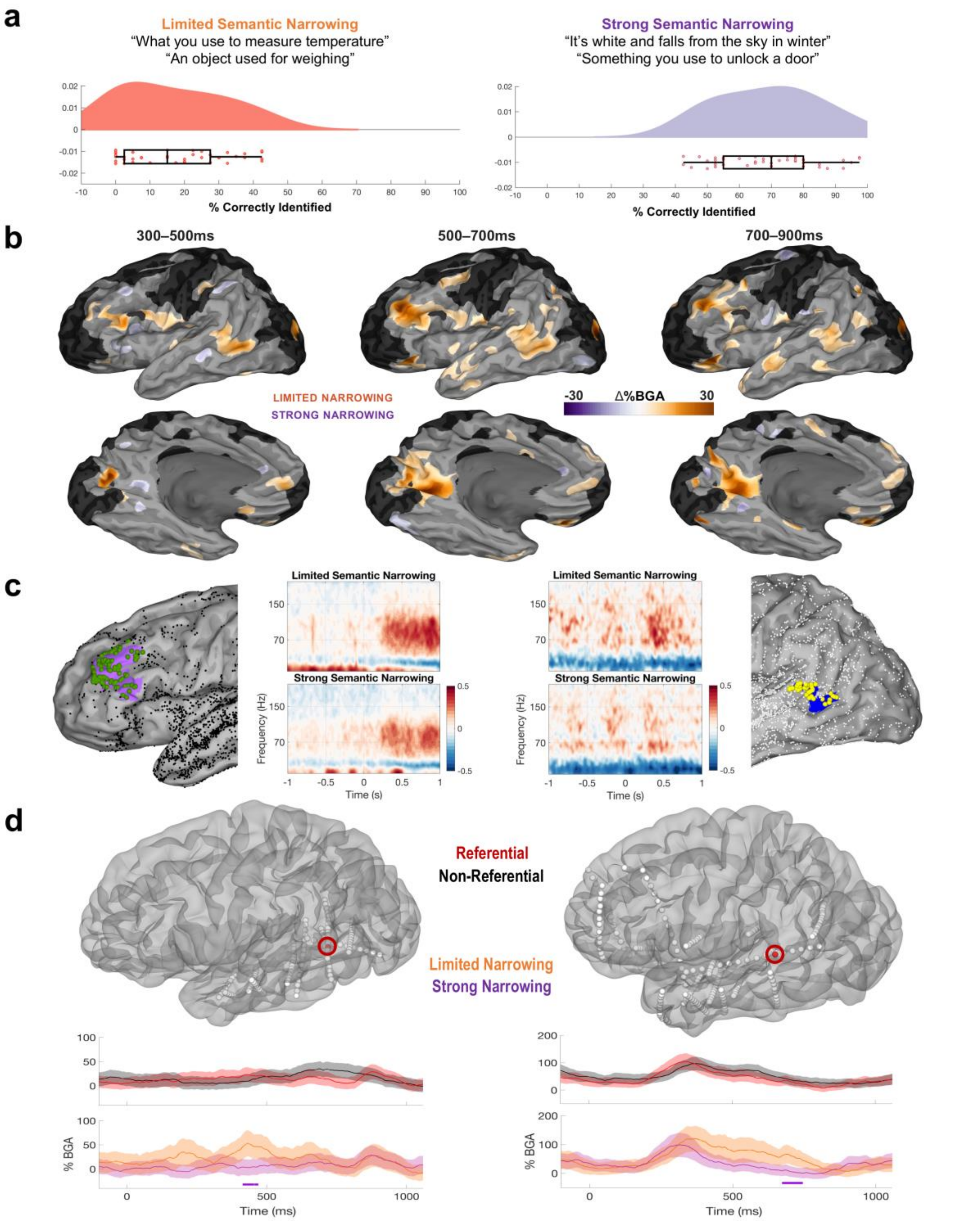

Supplementary Figure 5. **Cortical high gamma signatures of semantic narrowing. a** Percentage of participants in norming study who successfully defined the common object prior to the final word, grouping all trials (1-84 referential trials) into the top and bottom half percentages to derive the two groups. Generated using Raincloud[43]. Example trials are given above plots. **b** SB-MEMA contrasting limited narrowing (orange) and strong narrowing (purple) trials in BGA from 100-900 ms after final word onset (threshold: %BGA > 5%, t > 1.96, patient coverage ≥ 3, p < 0.01 corrected). **c** Left: Spectrogram and electrode placements for middle frontal gyrus and IFS (104 electrodes, 18 patients). Right: Spectrogram and electrode placements for posterior superior temporal sulcus

(pSTS) (electrodes: 46; patients: 21). **d** Exemplar electrode displaying greater broadband gamma activity for limited narrowing trials in pSTS, taken from two patients (left and right), contrasting with a lack of referential effect.
