# Supplementary material for: The spatiotemporal dynamics of semantic integration in the human brain
Source: Nat Commun. 2023 Oct 24;14:6336. doi: 10.1038/s41467-023-42087-8 (PMC10598228; doi:10.1038/s41467-023-42087-8)
Supplement: Supplementary file 2 — Reporting Summary [file 41467_2023_42087_MOESM2_ESM.pdf]

nature portfolio

Corresponding author(s): Nitin Tandon

Last updated by author(s): Aug 22, 2023

Reporting Summary

Nature Portfolio wishes to improve the reproducibility of the work that we publish. This form provides structure for consistency and transparency in reporting. For further information on Nature Portfolio policies, see our Editorial Policies and the Editorial Policy Checklist.

Statistics

For all statistical analyses, confirm that the following items are present in the figure legend, table legend, main text, or Methods section.

n/a	Confirmed	
☐	☒	The exact sample size (*n*) for each experimental group/condition, given as a discrete number and unit of measurement
☐	☒	A statement on whether measurements were taken from distinct samples or whether the same sample was measured repeatedly
☐	☒	The statistical test(s) used AND whether they are one- or two-sided<br>*Only common tests should be described solely by name; describe more complex techniques in the Methods section.*
☐	☒	A description of all covariates tested
☐	☒	A description of any assumptions or corrections, such as tests of normality and adjustment for multiple comparisons
☐	☒	A full description of the statistical parameters including central tendency (e.g. means) or other basic estimates (e.g. regression coefficient) AND variation (e.g. standard deviation) or associated estimates of uncertainty (e.g. confidence intervals)
☐	☒	For null hypothesis testing, the test statistic (e.g. F, t, r) with confidence intervals, effect sizes, degrees of freedom and P value noted<br>*Give P values as exact values whenever suitable.*
☒	☐	For Bayesian analysis, information on the choice of priors and Markov chain Monte Carlo settings
☒	☐	For hierarchical and complex designs, identification of the appropriate level for tests and full reporting of outcomes
☒	☐	Estimates of effect sizes (e.g. Cohen's *d*, Pearson's *r*), indicating how they were calculated

Our web collection on statistics for biologists contains articles on many of the points above.

Software and code

Policy information about availability of computer code

Data collection	Stimuli were presented using Psychophysics Toolbox (http://psychtoolbox.org/) run in MATLAB. Data were collected using the Neuroport recording system (Blackrock Microsystems, Salt Lake City, Utah).
Data analysis	Data were analyzed in MATLAB and through the following open source software packages: AFNI (http://afni.nimh.nih.gov/), Freesurfer (http://surfer.nmr.mgh.harvard.edu).

For manuscripts utilizing custom algorithms or software that are central to the research but not yet described in published literature, software must be made available to editors and reviewers. We strongly encourage code deposition in a community repository (e.g. GitHub). See the Nature Portfolio guidelines for submitting code & software for further information.

Data

Policy information about availability of data

All manuscripts must include a data availability statement. This statement should provide the following information, where applicable:

- Accession codes, unique identifiers, or web links for publicly available datasets
- A description of any restrictions on data availability
- For clinical datasets or third party data, please ensure that the statement adheres to our policy

The datasets generated from this research are not publicly available due to them containing information non-compliant with HIPAA and the human participants the data were collected from have not consented to their public release. However, they are available on request from the corresponding author.

Research involving human participants, their data, or biological material

Policy information about studies with human participants or human data. See also policy information about sex, gender (identity/presentation), and sexual orientation and race, ethnicity and racism.

Reporting on sex and gender	No sex or gender analyses were performed on the data, and we report sex below based on assigned at birth. Our paradigm concerned language processing common to all sexes and genders, so no further analyses were performed along these dimensions.
Reporting on race, ethnicity, or other socially relevant groupings	No factors such as race, ethnicity or socioeconomic status were used or were relevant to the present study.
Population characteristics	58 participants (11 male, 18-41 ± 5.7 years, IQ 95 ± 15, 2 left-handed, age of epilepsy onset 21 +/- 10 years) took part in the intracranial recording experiment after written informed consent was obtained. All participants were semi-chronically implanted with intracranial electrodes for the clinical purposes of seizure localization of pharmaco-resistant epilepsy.
Recruitment	Participants were recruited from the pool of patients scheduled to undergo intracranial monitoring at the Texas Comprehensive Epilepsy Program. Their participation in the study was voluntary, decoupled from their clinical care, and they were given the option of withdrawing participation and any time.
Ethics oversight	Committee for the Protection of Human Subjects at the University of Texas Health Science Center at Houston.

Note that full information on the approval of the study protocol must also be provided in the manuscript.

Field-specific reporting

Please select the one below that is the best fit for your research. If you are not sure, read the appropriate sections before making your selection.

☒ Life sciences ☐ Behavioural & social sciences ☐ Ecological, evolutionary & environmental sciences

For a reference copy of the document with all sections, see nature.com/documents/nr-reporting-summary-flat.pdf

Life sciences study design

All studies must disclose on these points even when the disclosure is negative.

Sample size	Given electrode placement in these patients was for clinical need rather than experimental purposes, the number of patients required was based on providing adequate coverage of the areas being studied, based on previous comparable studies. After data preprocessing, at least 60 trials per condition (per experimental block) were used for analysis from the naming to definition task.
Data exclusions	Inclusion criteria for this study were that the participants were English native speakers, left hemisphere dominant for language and did not have a significant additional neurological history (e.g., previous resections, MR imaging abnormalities such as malformations or hypoplasia).
Replication	Alongside population level effects in our patient cohort, in patients with sufficient coverage, we confirmed these effects within individual patients. Each experiment involved multiple trials (technical replicates) in multiple patients (biological replicates). Each patient received the same instructions before the task, and no patient was exposed to the stimuli prior to the task. Both individual (technical variability) and group results (biological variability) are shown. Results are consistent within and across patients.
Randomization	Patients were not assigned into experimental groups. Electrodes were grouped based on a brain parcellation from the Human Connectome Project.
Blinding	All patients were from the same cohort and consequently blinding was not possible.

Reporting for specific materials, systems and methods

We require information from authors about some types of materials, experimental systems and methods used in many studies. Here, indicate whether each material, system or method listed is relevant to your study. If you are not sure if a list item applies to your research, read the appropriate section before selecting a response.

Materials & experimental systems

n/a	Involved in the study
☒	☐ Antibodies
☒	☐ Eukaryotic cell lines
☒	☐ Palaeontology and archaeology
☒	☐ Animals and other organisms
☒	☐ Clinical data
☒	☐ Dual use research of concern
☒	☐ Plants

Methods

n/a	Involved in the study
☒	☐ ChIP-seq
☒	☐ Flow cytometry
☒	☐ MRI-based neuroimaging
